# Supplementary material for: Nsun2 coupling with RoRγt shapes the fate of Th17 cells and promotes colitis
Source: Nat Commun. 2023 Feb 16;14:863. doi: 10.1038/s41467-023-36595-w (PMC9932167; doi:10.1038/s41467-023-36595-w)
Supplement: Supplementary file 1 — Supplementary Information [file 41467_2023_36595_MOESM1_ESM.pdf]

Supplementary Information

Nsun2 coupling with RoRγt shapes the fate of Th17 cells and promotes colitis

Wen-Lan Yang, Weinan Qiu, Ting Zhang, Kai Xu, Zi-Juan Gu, Yu Zhou, Heng-Ji Xu, Zhong-Zhou Yang, Bin Shen, Yong-Liang Zhao, Qi Zhou, Ying Yang, Wei Li, Peng-Yuan Yang, Yun-Gui Yang

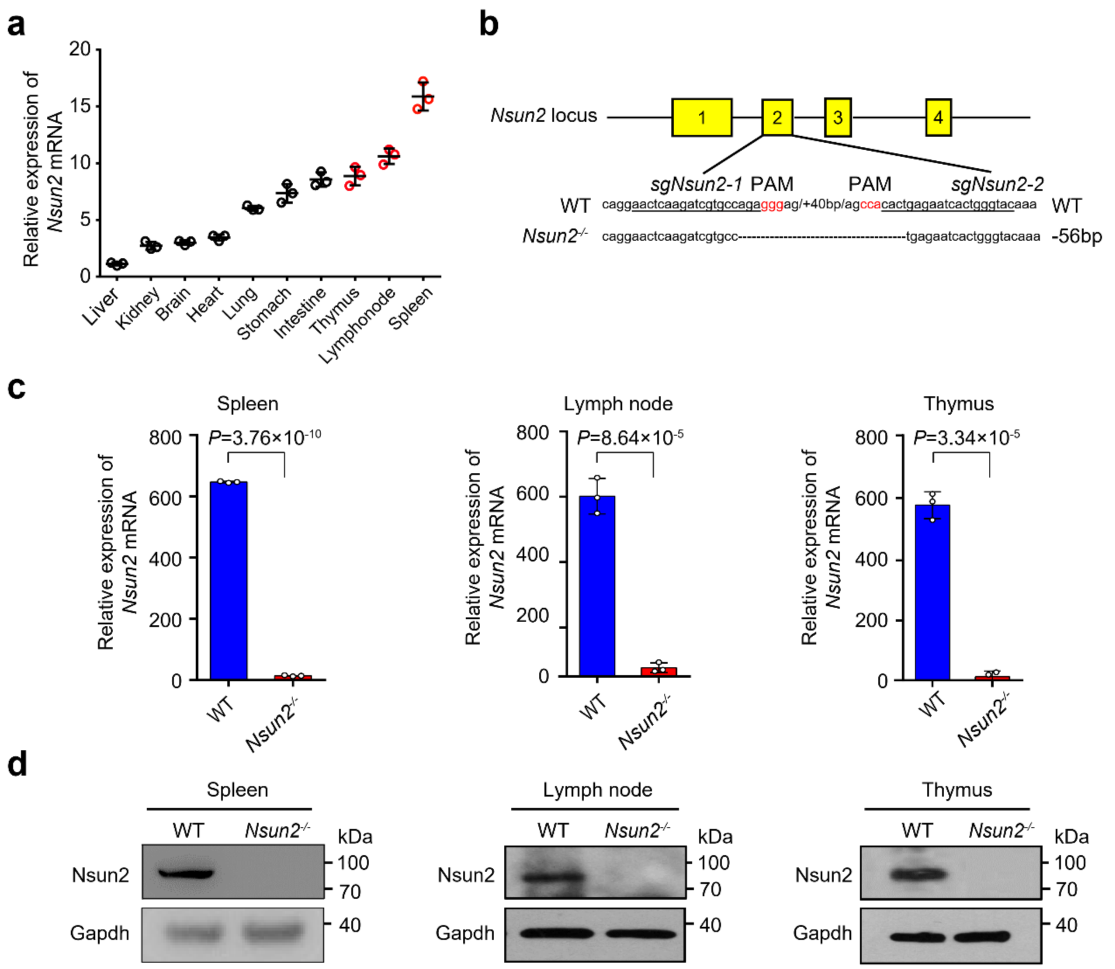

Supplementary Fig. 1 | The expression of Nsun2 immune organs and validation of Nsun2 knockout mice.

a, The mRNA level of *Nsun2* in mouse organs were detected by RT-qPCR ($n = 3$ per group). **b**, Strategies for construction of Nsun2 knockout mice. **c**, **d**, The knockout efficiency of Nsun2 was detected. Both RNA and protein were extracted from the spleen, lymph node, and thymus of wild-type and *Nsun2*$^{-/-}$ mice respectively, and then carried out RT-qPCR ($n = 3$ per group) (**c**) and western blot (**d**) to detect Nsun2 expression. Data are representative of two independent experiments. *P* values are calculated by using two-tailed unpaired Student's *t*-test. Error bars represent mean ± s.e.m (**a, c**). Source data are provided as a Source Data file.

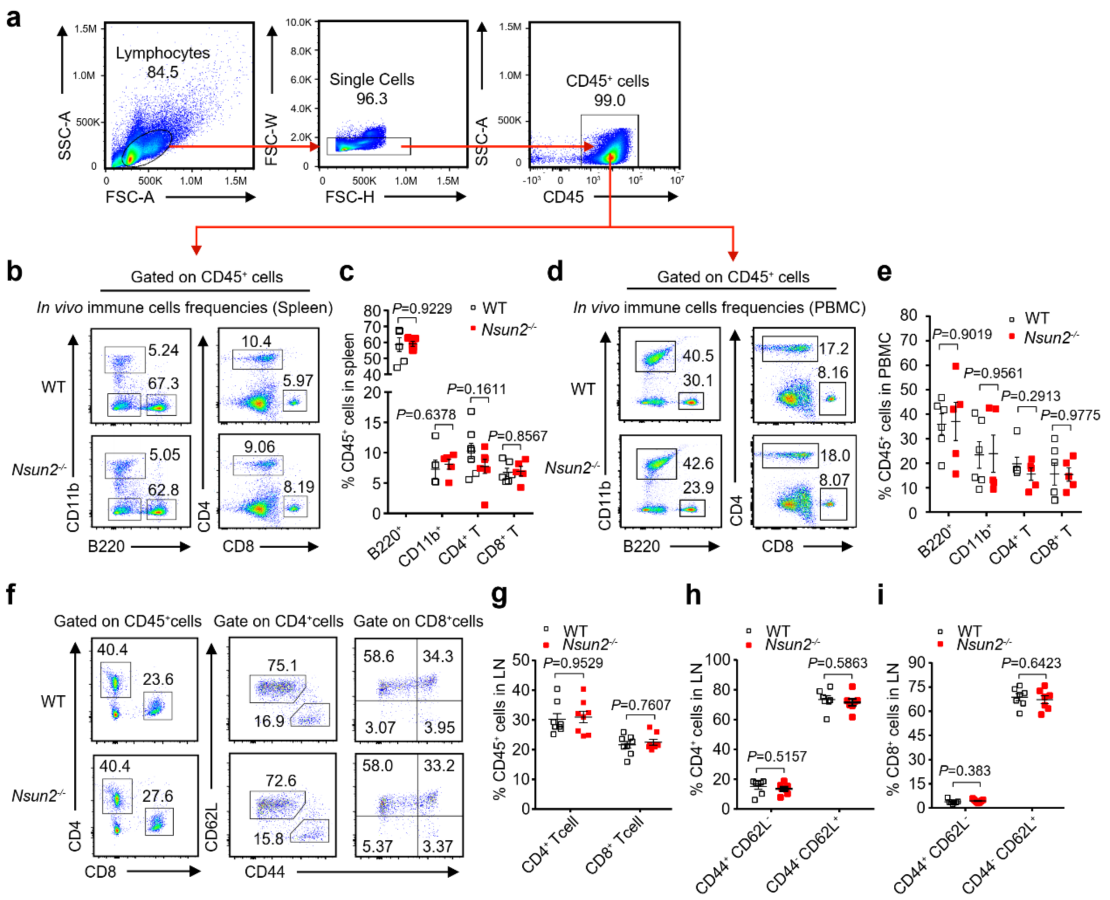

Supplementary Fig. 2 | Immunophenotype analysis of wild-type and *Nsun2*$^{-/-}$ mice.

a. Gating strategy to Supplementary Fig. 2b-i. **b-e**, The T cell linages frequencies of B220^{+}, CD11b^{+}, CD4^{+} and CD8^{+} cells in spleen (**b, c**) and peripheral blood mononuclear cell (PBMC) (**d, e**) of littermate wild-type and *Nsun2*$^{-/-}$ mice were analyzed by FACS. Summary of the frequency of each cells are shown in **c** (n = 6 for wild-type and n = 5 for *Nsun2*$^{-/-}$ in B220^{+} cells and CD8^{+} cells; n = 5 for wild-type and *Nsun2*$^{-/-}$ in CD11b^{+} cells; n = 8 for wild-type and n = 7 for *Nsun2*$^{-/-}$ in CD4^{+} cells) and **e** (n = 6 for wild-type and n = 5 for *Nsun2*$^{-/-}$ in each cells). **f-i**, The cell frequencies of CD4^{+} cells, CD8^{+} cells, naïve T cells and activated T cells in lymph nodes of wild-type and *Nsun2*$^{-/-}$ mice were analyzed by FACS. Summary of the frequency of each cells are shown in **g-i** (n = 7 per group). All data are two independent experiments. P values are calculated by using two-tailed unpaired Student's t-test. Error bars represent mean ± s.e.m (**c, e, g-i**). Source data are provided as a Source Data file.

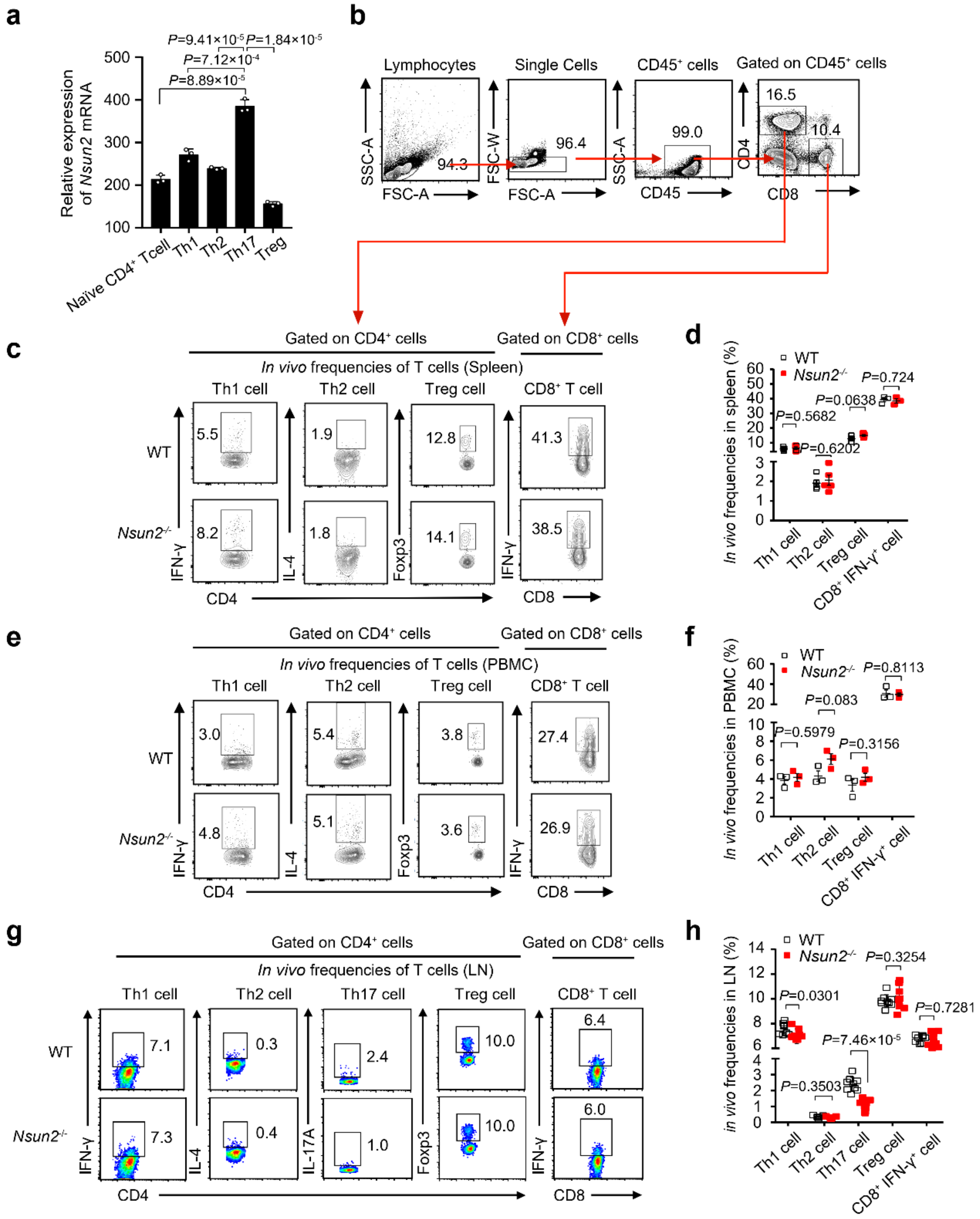

Supplementary Fig. 3 | Nsun2 is critical for Th17 cells differentiation *in vivo*.

a, The mRNA expression level of *Nsun2* in naïve CD4^{+} T cells, Th1, Th2, Th17, and Treg cells were detected by RT-qPCR (n = 3 per group). Sorted naïve CD4^{+} T cells from lymph nodes of wild-type C57BL/6 mice were differentiated under Th1, Th2, Th17 and Treg inducing conditions respectively, and total RNA were then isolated from each T cell subsets. **b**, Gating strategy related to Fig. 1c-d and Supplementary Fig. 3c-h. **c, d,** The percentage of CD4^{+} IFN-γ^{+} Th1, CD4^{+} IL-4^{+} Th2, CD4^{+} FOXP3^{+} Treg and CD8^{+} IFN-γ^{+} cells in spleen from littermate wild-type and *Nsun2*$^{-/-}$ mice were analyzed by FACS. Summary of the frequency of each cells are shown in **d** (n = 5 for wild-type and *Nsun2*$^{-/-}$ in Th1, Th2 and Treg cells; n = 3 for wild-type and *Nsun2*$^{-/-}$ in CD8^{+} IFN-γ^{+} cells). **e, f,** The percentage of CD4^{+} IFN-γ^{+} Th1, CD4^{+} IL-4^{+} Th2,

$CD4^+$ $FOXP3^+$ Treg and $CD8^+$ IFN-γ^+ cells in PBMC from littermate wild-type and *Nsun2*$^{-/-}$ mice were analyzed by FACS. Summary of the frequency of each cells are shown in **f** (*n* = 3 per group). **g**, **h**, The percentage of $CD4^+$ IFN-γ^+ Th1, $CD4^+$ IL-4^+ Th2, $CD4^+$ IL-$17A^+$ Th17, $CD4^+$ $FOXP3^+$ Treg and $CD8^+$ IFN-γ^+ cells in lymph node from littermate wild-type and *Nsun2*$^{-/-}$ mice were analyzed by FACS. Summary of the frequency of each cells are shown in **h** (*n* = 9 per group). *P* values are calculated by using two-tailed unpaired Student's *t*-test. Error bars represent mean ± s.e.m (**a, d, f and h**). Source data are provided as a Source Data file.

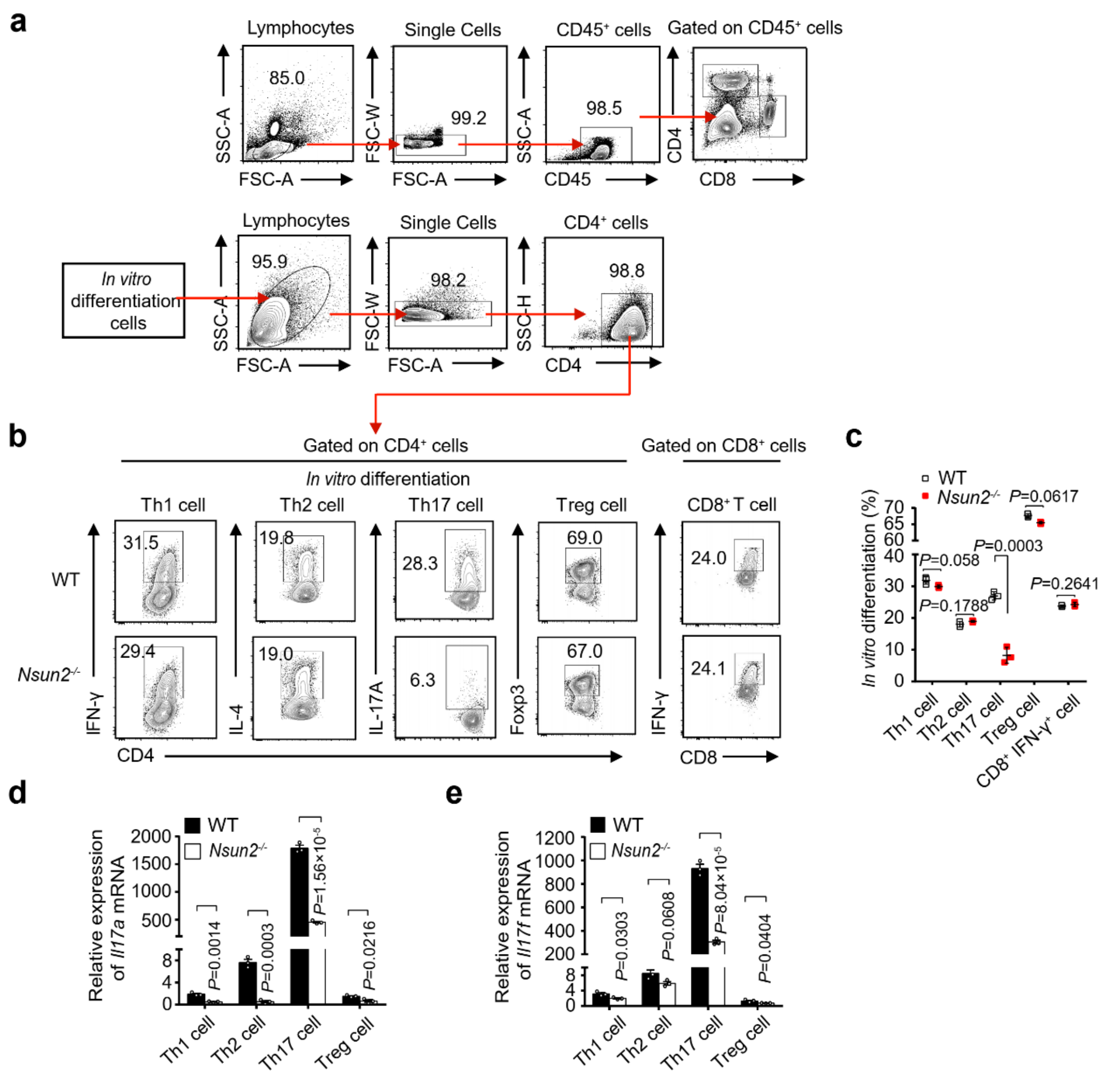

Supplementary Fig. 4 | Nsun2 is critical for Th17 cells differentiation *in vitro*.

a. Gating strategy related to Fig. 1e-f and Supplementary Fig. 4b-c. **b**, **c**, The *in vitro* differentiation frequencies of Th1, Th2, Treg, Th17 and CD8$^+$ T cells in wild-type and *Nsun2*$^{-/-}$ mice were analyzed by FACS. Summary of the frequency of each cells are shown in **c** (n = 3 per group). **d**, **e**, The mRNA level of *Il17a* and *Il17f* in littermate wild-type and *Nsun2*$^{-/-}$ T subsets were detected by RT-qPCR (n = 3 per group). All data are at least two independent experiments. P values are calculated by using two-tailed unpaired Student's t-test. Error bars represent mean ± s.e.m (**c-e**). Source data are provided as a Source Data file.

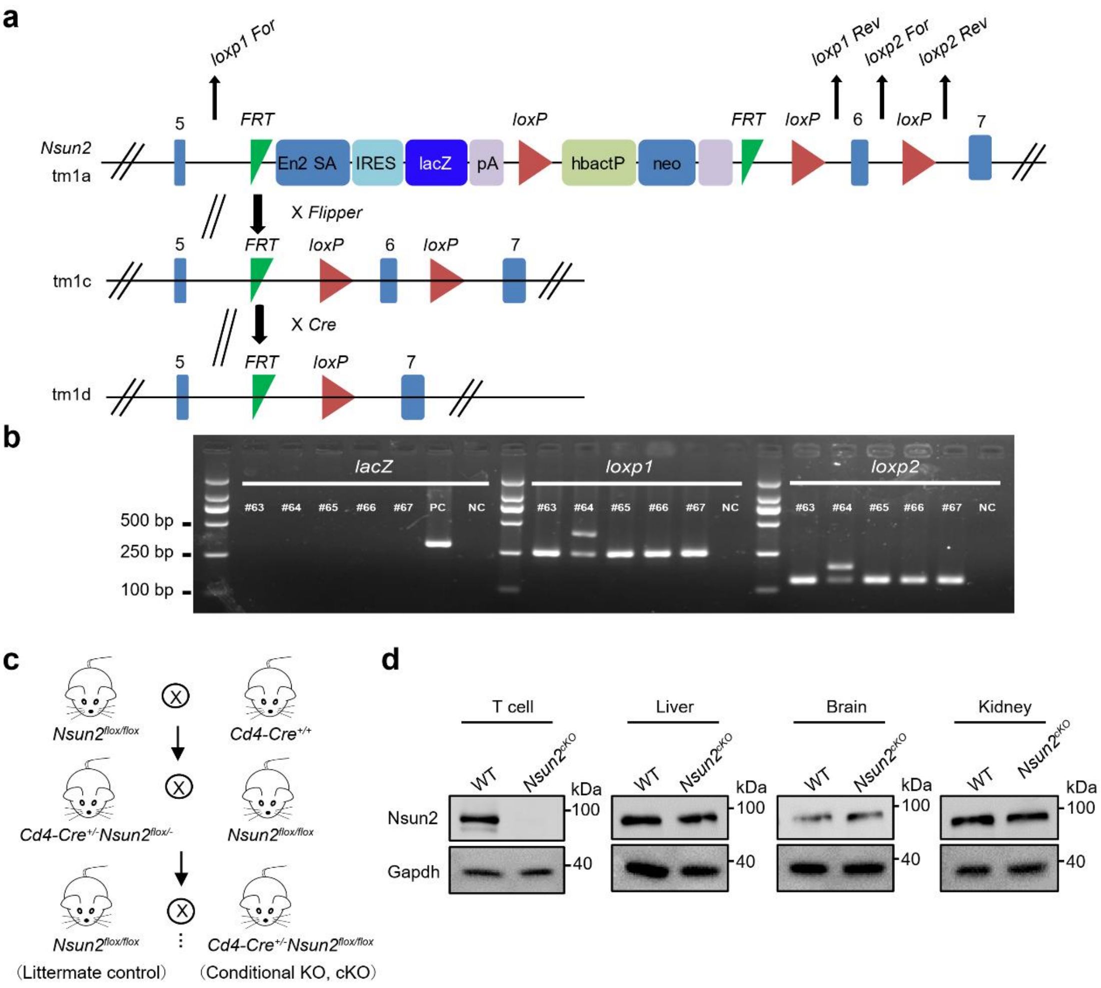

Supplementary Fig. 5 | Strategies for construction of *Nsun2*^{cKO} mice.

a, Schema for gene targeting to generate floxed *Nsun2* mice, the KO first mice (tm1a) were mated with FLPeR mice to delete the *Neo* cassette to generate *Nsun2*-floxed mice (tm1c), *Nsun2* conditional KO mice should be generated by crossing with *cre* mice (tm1d). **b**, Genotyping for generating *Nsun2* floxed mice. The heterozygous floxed mice (#64) was typed by two pairs of primers: *loxp1*(f1: 391 bp, WT: 251 bp); *loxp2*(f2: 182 bp, WT: 128 bp) versus positive control (PC) and negative control (NC). **c**, Strategy for construction of *Nsun2*$^{f/f}$; *Cd4-Cre*$^{+/-}$ mice. **d**, Determine conditional knockout of Nsun2 in T cells sorting from lymph nodes in wild-type or *Nsun2*^{cKO} mice by western blot, and the Nsun2 protein expression in liver, brain and kidney as the control.

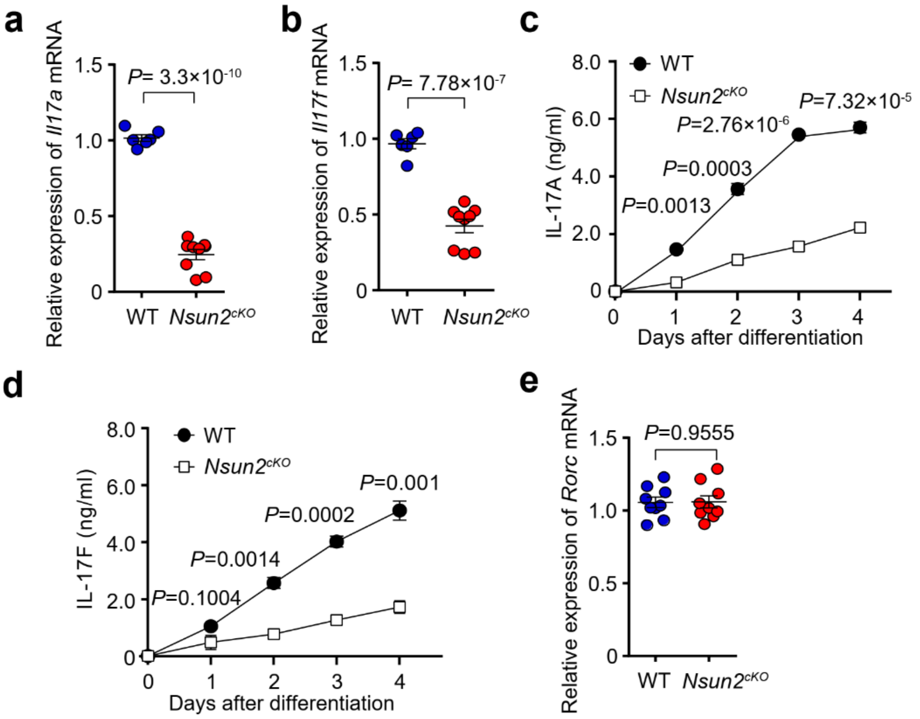

Supplementary Fig. 6 | T cell-specific Nsun2 deletion impairs Th17 generation.
a, b, The mRNA expression of *Il17a* (**a**) and *Il17f* (**b**) in wild-type and *Nsun2^cKO^* Th17 cells were detected by RT-qPCR (n = 6 for wild-type; n = 9 for *Nsun2^cKO^*). **c, d,** The secretions of IL-17A (**c**) and IL-17F (**d**) was detected by ELISA. The supernatants were collected from wild-type and *Nsun2^cKO^* Th17-inducing media as indicated time points (0-4 days) (n = 3 per group). **e**, The mRNA expression of *Rorc* in Th17 cells was detected by RT-qPCR (n = 9 per group). All data are at least two independent experiments. *P* values are calculated by using two-tailed unpaired Student's *t*-test. Error bars represent mean ± s.e.m (**a-e**). Source data are provided as a Source Data file.

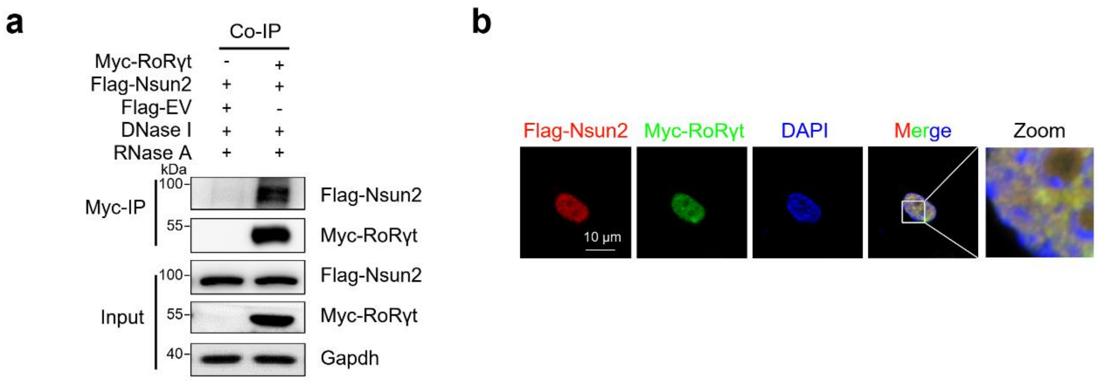

Supplementary Fig. 7 | Nsun2 reprograms Th17 features *via* direct interaction with RoRγt.

a, Co-immunoprecipitation showing the interaction of Nsun2 and RoRγt *in vitro*. HEK293T cells were transfected with Myc-RoRγt and Flag-Nsun2 or Flag-EV (as control) plasmids for 2 days. Cell lysates were collected and treated with DNase I and RNase A before anti-Myc-immunoprecipitation. **b**, Immunofluorescence showing the interaction of Flag-Nsun2 and Myc-RoRγt in HEK293T cells. HEK293T cells were transfected with Myc-RoRγt and Flag-Nsun2 plasmids for 24 hours, and then were subjected to immunofluorescence assay. All data are representative of two independent experiments. Source data are provided as a Source Data file.

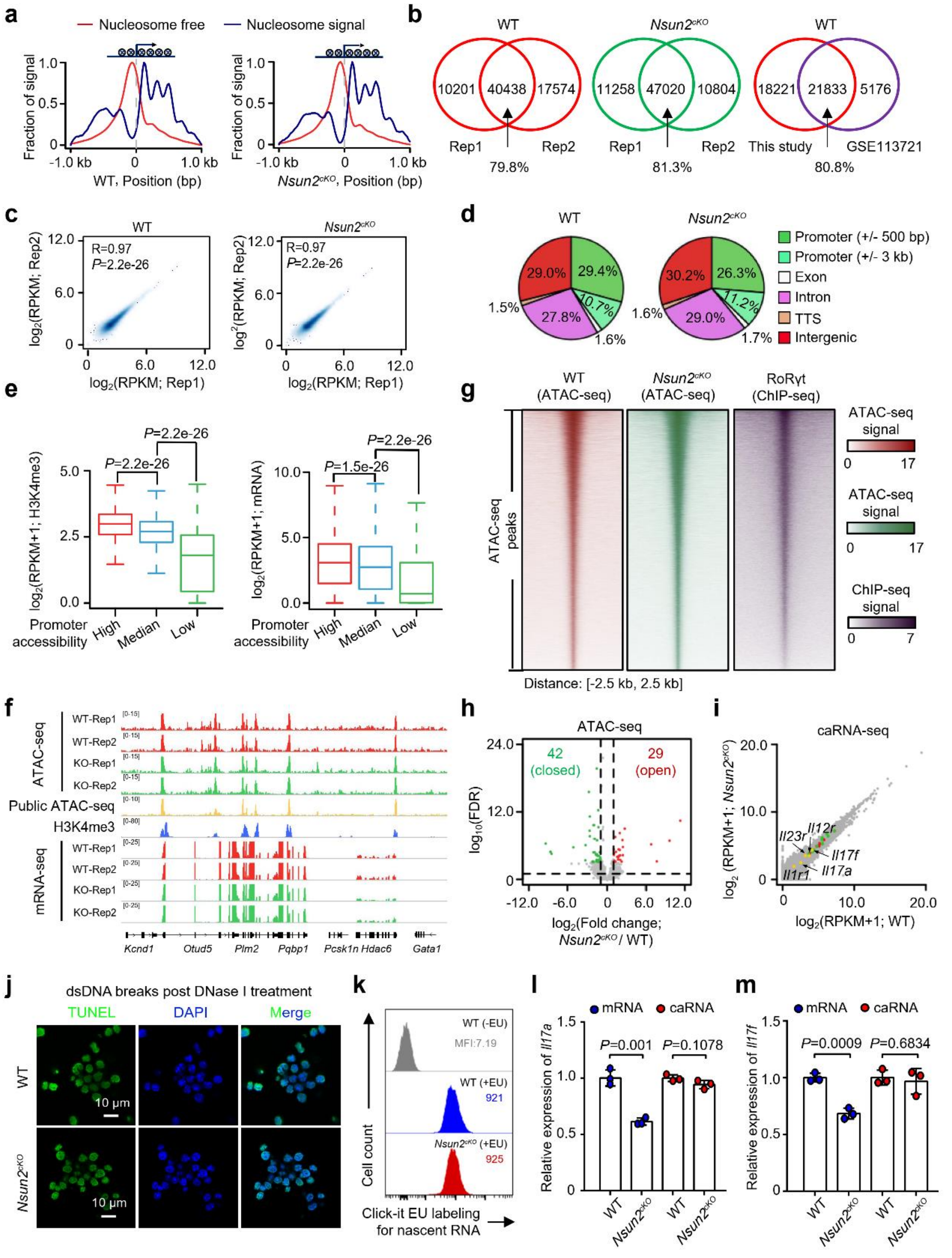

Supplementary Fig. 8 | Nsun2 deficiency does not dampen the chromatin state and transcription initiation.

a, Footprints of nucleosome around TSSs using ATAC-seq of wild-type and *Nsun2*^{cKO} Th17 cells. **b**, Venn plots showing the overlap of ATAC-seq peaks between two replicates of wild-type and *Nsun2*^{cKO} Th17 cells, as well as the overlap of ATAC-seq peaks detected in wild-type Th17 cells for this study and GSE113721. **c**, Scatter plots showing the repeatability of the ATAC-seq enrichment between two replicates of wild-type and *Nsun2*^{cKO} Th17 cells. *P* values are calculated by using two-sided student's *t*-test. **d**, Genome-wide distribution of peaks for

wild-type and *Nsun2^{cKO}* Th17 cells identified by MACS2. **e**, Box plots showing promoter enrichment of H3K4me3 (left; GSE40918) and gene expression (right; mRNA-seq) in wild-type Th17 cell for genes with different promoter enrichment of ATAC-seq. Only the top 15K genes with highest promoter enrichment of ATAC-seq were used and then were divided into three equal groups in this analysis. *P* values were determined by two-way Mann-Whitney U test and only value between Q1-1.5*IQR and Q3+1.5*IQR has been shown in boxplot. **f,** IGV tracks showing the ATAC-seq, H3K4me3, and mRNA-seq signal in Th17 cells. **g**, ATAC-seq summit-centered heatmap of ATAC-seq signal in wild-type and *Nsun2^{cKO}*, as well as RORγt ChIP-seq signal (GSE40918) in the same regions for all ATAC-seq peaks. **h**, Volcano plot showing the open (in Red) and closed (in Green) ATAC-seq peaks upon Nsun2 deficiency in Th17 cells. **i**, Scatter plot of expression of caRNAs in wild-type compared to *Nsun2^{cKO}* Th17 cells. Part of important genes involving in T cell receptor signaling pathway (in Red), T cell differentiation (in Green) and cytokines (in Gold) were also shown. **j**, TUNEL assay to detect the global chromatin accessibility in wild-type and *Nsun2^{cKO}* Th17 cells. **k**, Click-it EU labeling and FACS analysis to detect global nascent RNA level in wild-type and *Nsun2^{cKO}* Th17 cells. **l**, **m**, RT-qPCR showing the expression of *Il17a* and *Il17f* on caRNA and mRNA level (n = 3 per group). All data are two independent experiments. *P* values are calculated by using two-tailed unpaired Student's *t*-test. Error bars represent mean ± s.e.m (**l, m**). Source data are provided as a Source Data file.

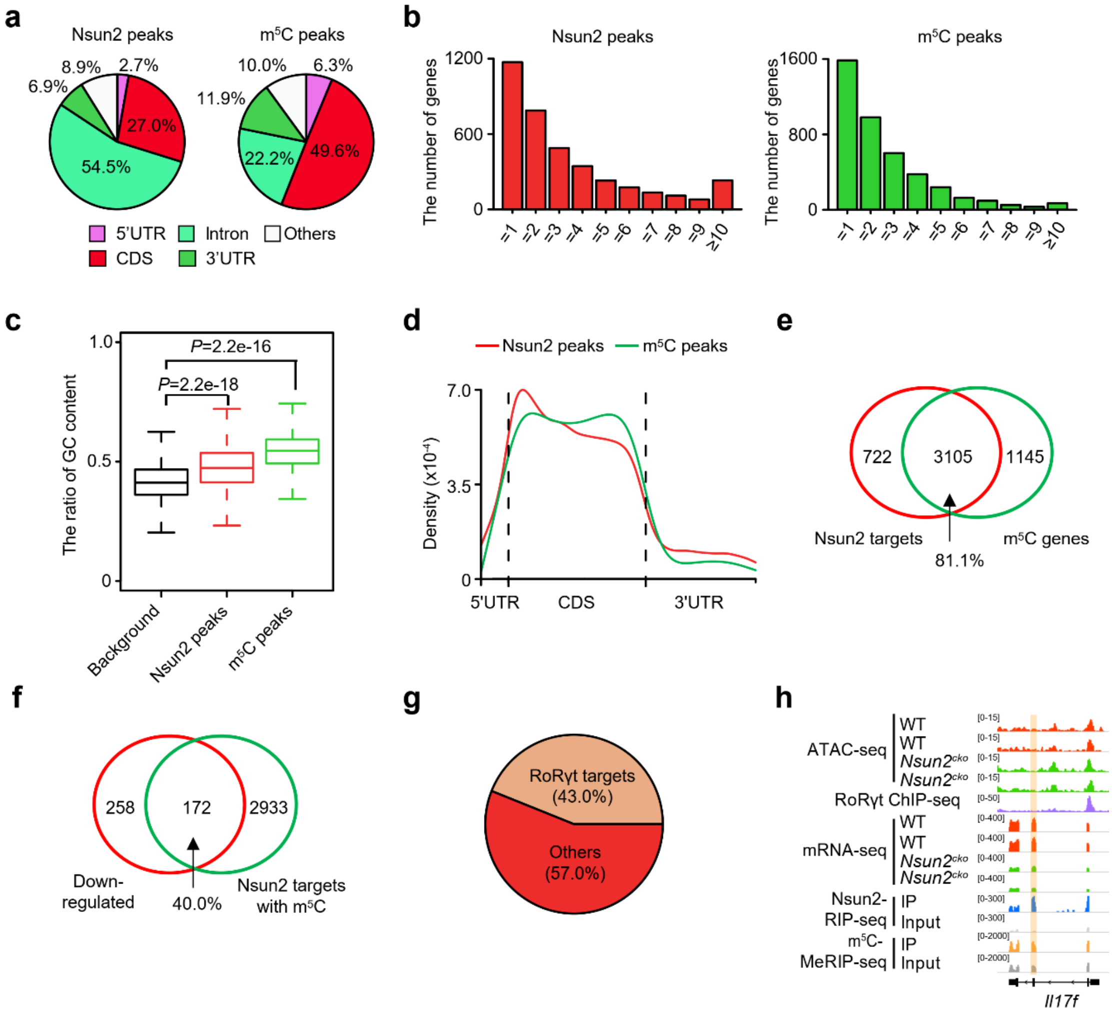

Supplementary Fig. 9 | Quality control of Nsun2 RIP-seq and m^{5}C MeRIP-seq.

a, Pie diagrams showing the ratio of Nsun2 (left) and m^{5}C peaks (right) for ncRNAs or different segments of mRNAs. **b**, Bar plots showing numbers of genes with different number of Nsun2 (left) and m^{5}C peaks (right). **c**, Box plots displaying the variation of GC ratio containing in all random (in Black), Nsun2 (in Red) and m^{5}C peaks (in Green). *P* values were determined by two-way Mann-Whitney U test and only value between Q1-1.5*IQR and Q3+1.5*IQR has been shown in boxplot. **d**, The distribution of Nsun2 (in Red) and m^{5}C peaks (in Green) along mRNA 5'UTR, CDS and 3'UTR. **e**, Venn plot showing the overlay between Nsun2 targets and m^{5}C genes. **f**, Venn diagram displaying the overlay between down-regulated genes and Nsun2 targets with m^{5}C. **g**, Pie plot showing the ratio of RORγt targets among the genes which were Nsun2 targets with m^{5}C and were also down-regulated upon Nsun2 depletion. **h**, IGV tracks displaying ATAC-seq, RoRγt ChIP-seq (GSE40918), mRNA-seq, Nsun2 RIP-seq (IP and input) and m^{5}C MeRIP-seq (IP and input) enrichment near *Il17f*. All data are representative of two independent experiments.

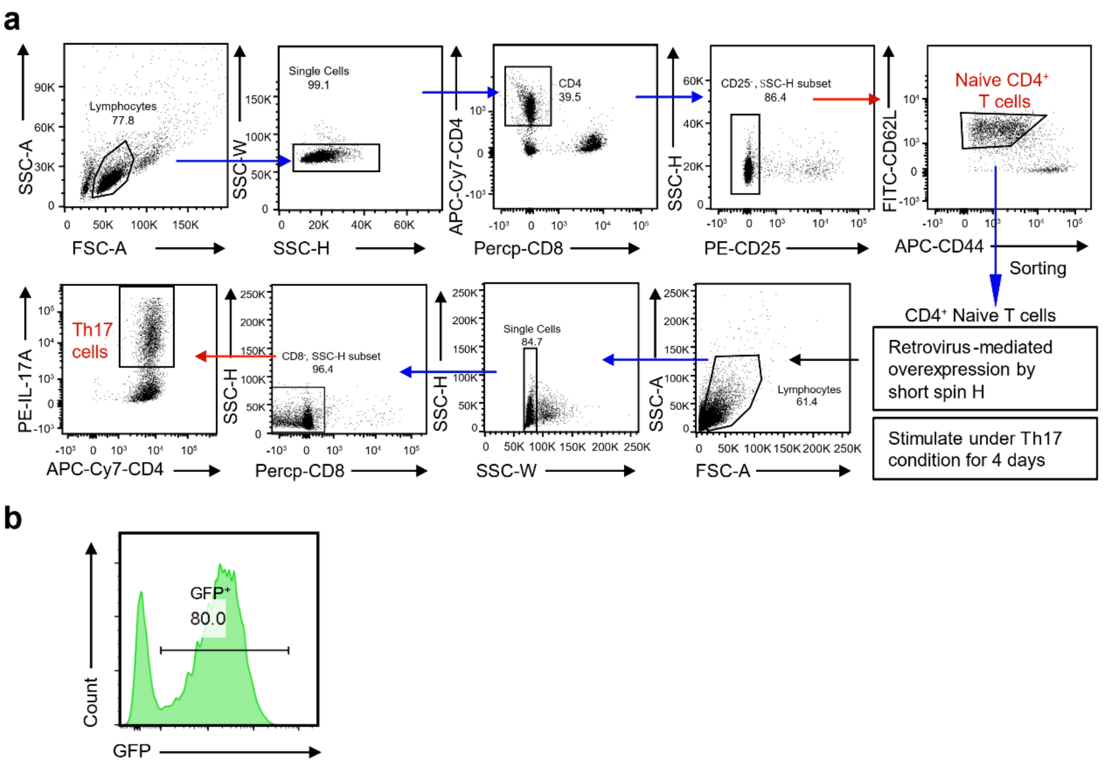

Supplementary Fig. 10 | Nsun2-mediated m^{5}C modification is required for Th17 generation.

a, Gating strategy for retrovirus-mediated gene overexpression and Th17 *in vitro* differentiation, and related to Fig. 3e and f. **b**, Flow cytometry analysis showed the infected efficiency of retrovirus in Th17 cells. Data shown is representative of four independent experiments.

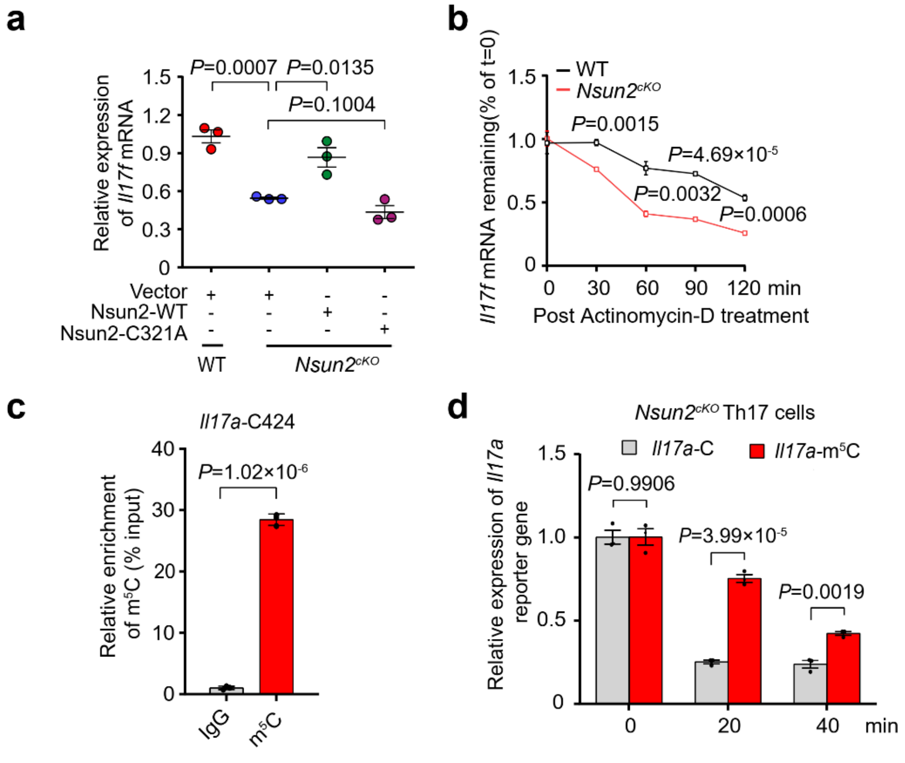

Supplementary Fig. 11 | Nsun2 controls Th17 associated mRNA stability through marking with m^{5}C modification.

a, The mRNA level of *Il17f* in Th17 cells of rescuing Nsun2-WT, Nsun2-C321A, and Vector in were detected by RT-qPCR (n = 3 per group). **b**, The half-lives of *Il17f* in wild-type and *Nsun2^{cKO}* were detected by RT-qPCR (n = 3 per group). Sorted naïve CD4$^+$ T cells from wild-type and *Nsun2^{cKO}* mice respectively were induced under Th17-inducing condition for 4 days, and inhibiting RNA transcription in Th17 cells by treating with Actinomycin D, and harvesting the cells as indicated times (0, 20, and 40 min). The residual RNAs were normalized to 0 min. **c**, m^{5}C MeRIP-qPCR analysis results showing the 424 cytosine site of *Il17a* marks m^{5}C modification (n = 3 per group). **d**, The RNA abundance of the *Il17a*-m^{5}C (in Red) and *Il17a*-C (in Black) reporter in *Nsun2^{cKO}* Th17 cells were detected by RT-qPCR and normalized to the value at 0 min (n = 3 per group). Gapdh served as an internal RNA control. All data are two independent experiments. *P* values are calculated by using two-tailed unpaired Student's *t*-test (**a-d**). Error bars represent mean ± s.e.m. Source data are provided as a Source Data file.

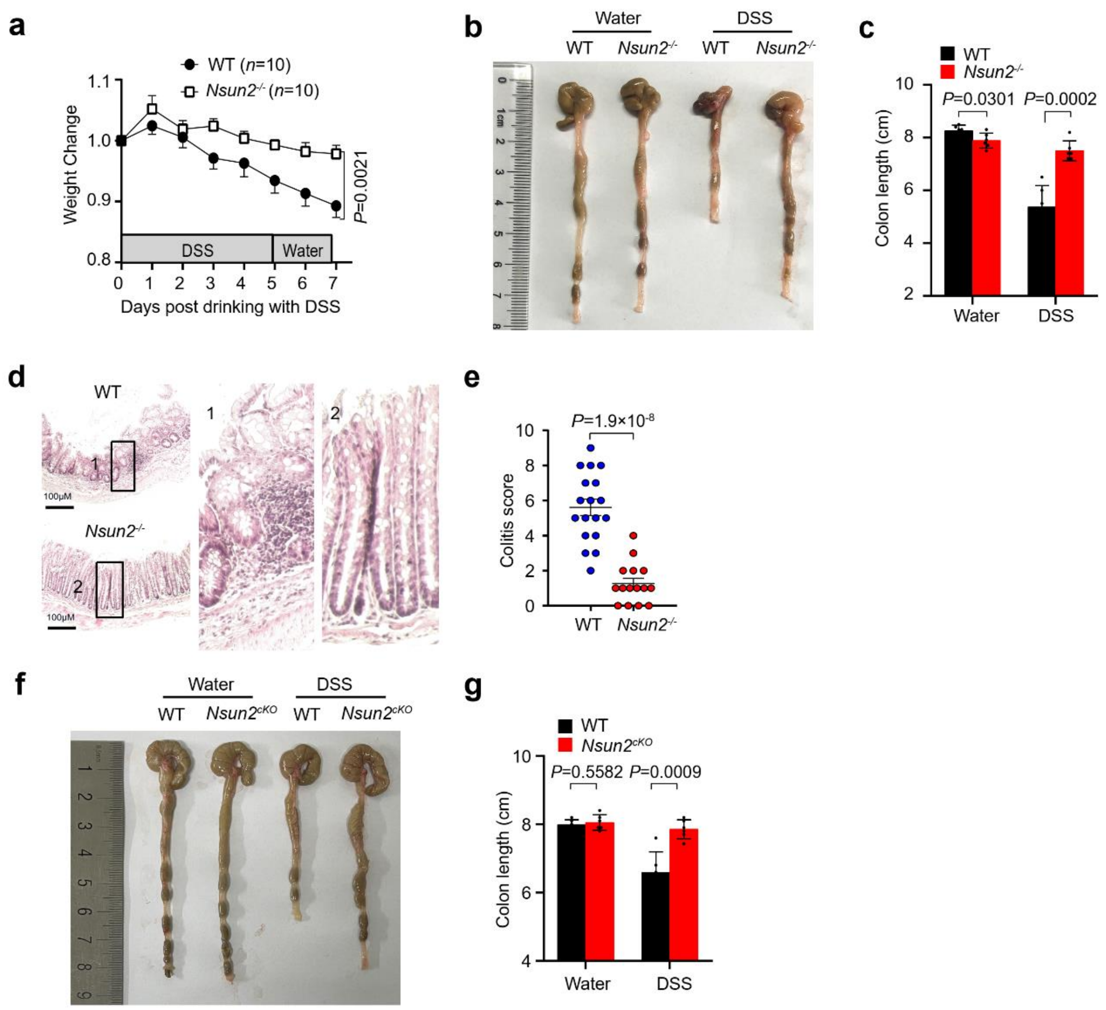

Supplementary Fig. 12 | Nsun2 deficiency ameliorates inflammations.

a-e, The phenotype of wild-type and *Nsun2*$^{-/-}$ mice under DSS-induced. Littermate wild-type and *Nsun2*$^{-/-}$ mice were treated with 3% DSS in drinking water. The body weight changes (**a**), representative macrograph of colons (**b**), summary of colon length (n = 6 per group) (**c**), hematoxylin-eosin staining indicated the severity of colons damage and inflammatory infiltration (Scale bar, 100 μm) (**d**), and colitis score (n = 18 images for WT and n = 15 images *Nsun2*$^{-/-}$) (**e**). **f**, **g**, The representative macrograph of colons compared between wild-type and *Nsun2*^{cKO} mice before or after DSS treatment, and summary of colon length (n = 6 per group) (**g**). All data are at least two independent experiments. *P* values are calculated by two-tailed unpaired Student's *t*-test (**a**, **c, e and g**). Error bars represent mean ± s.e.m. Source data are provided as a Source Data file.

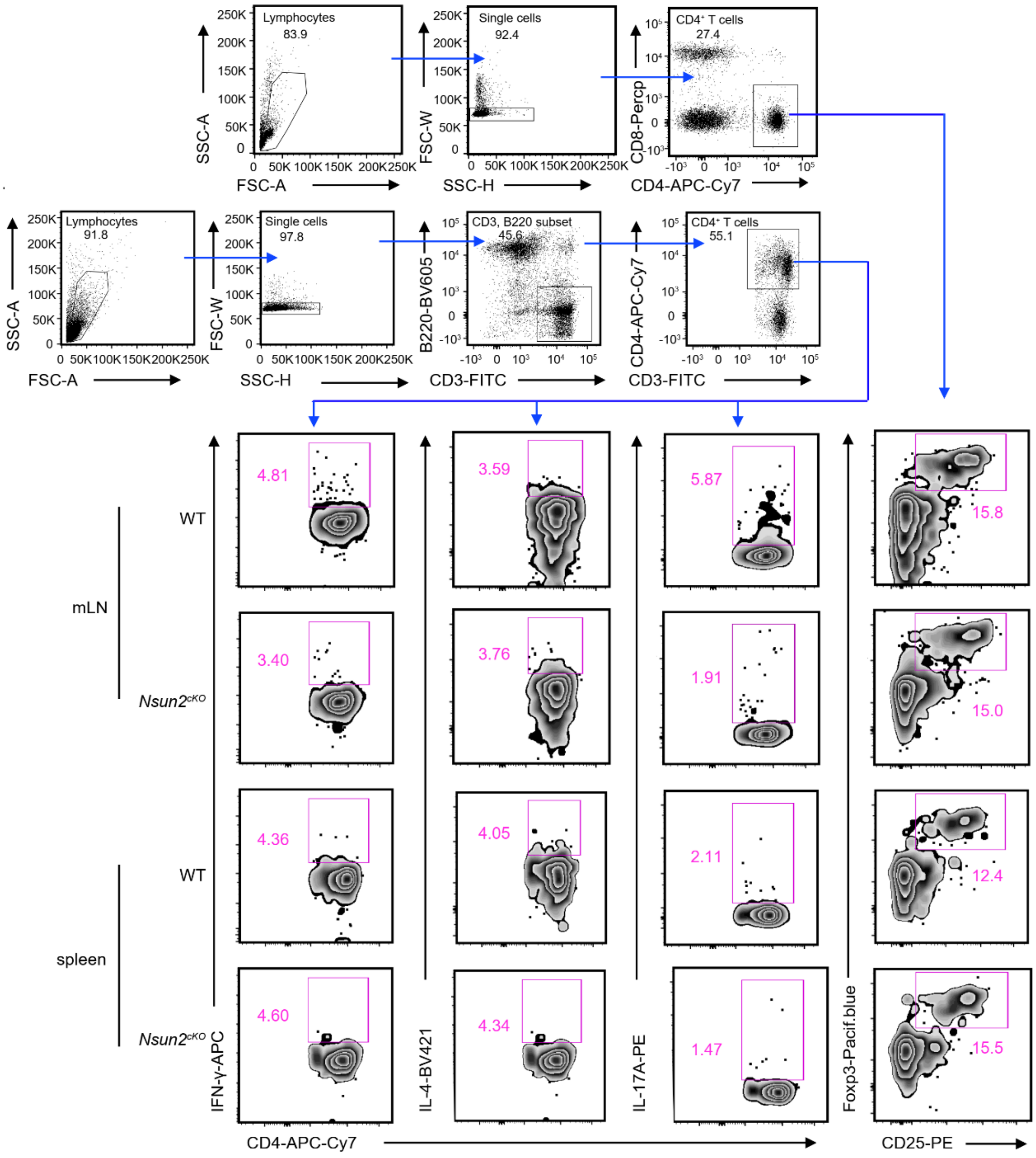

Supplementary Fig. 13 | Nsun2 deficiency ameliorates DSS-induced colitis.

Gating strategies related to Fig. 4d-g. The flow cytometry analysis of T subsets in mesenteric lymph nodes and spleen.

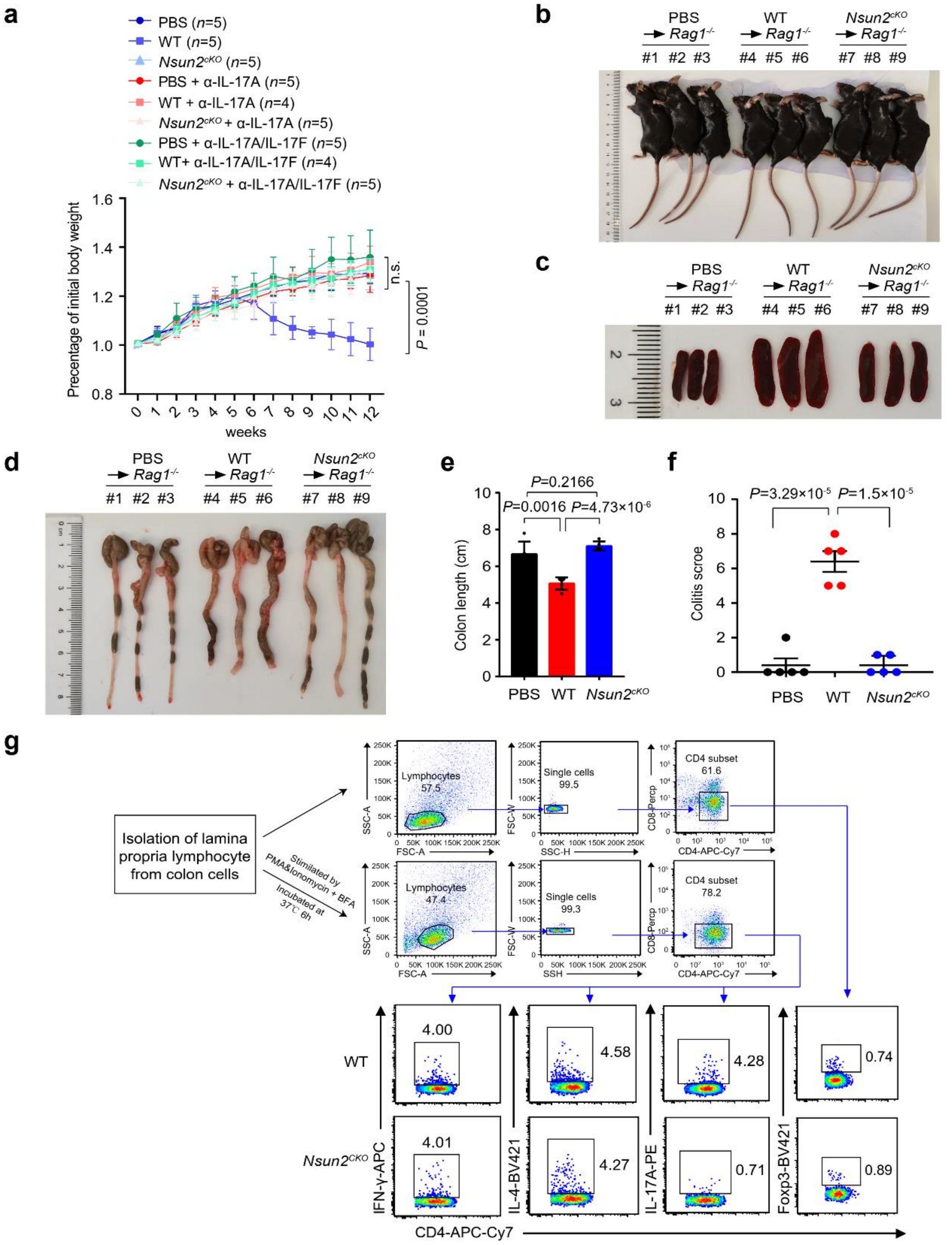

Supplementary Fig. 14 | *Nsun2^{cKO}* naïve T cells does not promote disease in CD45RB^{hi} adoptive transfer colitis mouse model.

a, The body weight changes of *Rag1$^{-/-}$* host mice transferred with wild-type and *Nsun2^{cKO}* naïve CD45RB^{hi} T cell, followed by the treatment (two times per week) with neutralizing antibody of IL-17A alone or IL-17A plus IL-17F. **b-h,** The phenotype of wild-type (n = 5) and *Nsun2^{cKO}* (n = 5) naïve T cell adoptively transferring into *Rag1$^{-/-}$* recipient mice. The representative macrograph of mice (**b**), representative image of spleens (**c**) and colons (**d**), colon length (n = 5 per group) (**e**), colitis score (n = 5 per group) (**f**). **g**. The representative FACS profiles of IFNγ^{+}

$CD4^+$ T, IL-4^+ $CD4^+$ T, IL-$17A^+$ $CD4^+$ T and $Foxp3^+$ $CD4^+$ T cells frequency in colonic lamina propria $CD4^+$ T cells from recipient mice transferring $CD4^+CD25^-CD45Rb^{hi}$ naïve T cells of wild-type or *Nsun2*^{cKO} mice (Gating strategy related to Fig. 4k). PBS vehicle injection was used as control. n = number of biological replicates. P values are calculated by using two-tailed unpaired Student's t-test (**a**, **e and f**). Error bars represent mean ± s.e.m. Source data are provided as a Source Data file.

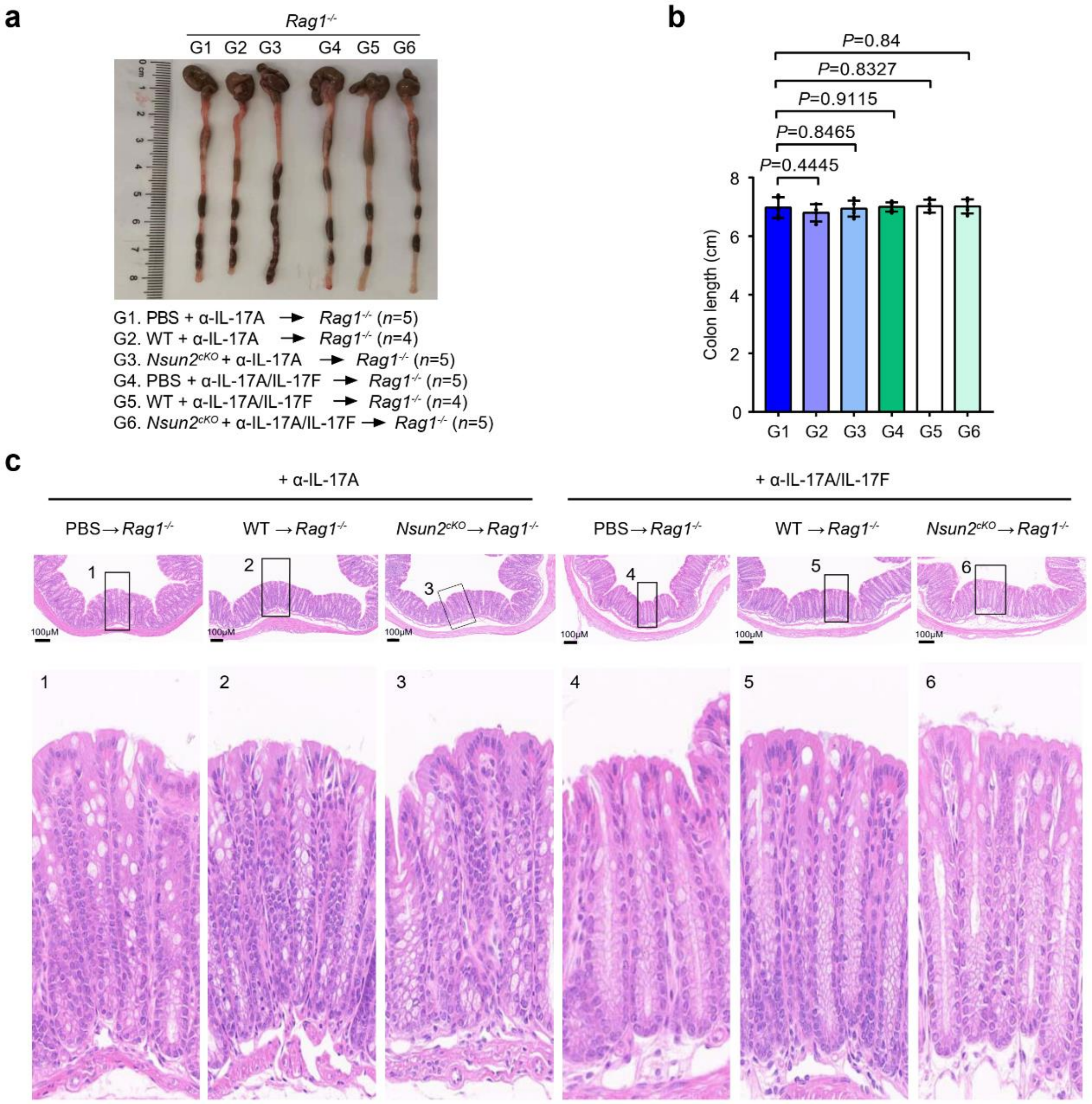

Supplementary Fig. 15 | Blockage of IL-17A or both IL-17A and IL-17F signaling eliminates colitis development in CD45RB^{hi} adoptive transfer colitis.

a-c, The phenotype of *Rag1*$^{-/-}$ host mice transferred with wild-type and *Nsun2*^{cKO} naïve CD45RB^{hi} T cell, followed by the treatment (two times per week) with neutralizing antibody of IL-17A alone or IL-17A plus IL-17F. The representative macrograph of colons (**a**), colon length (**b**), and hematoxylin-eosin staining indicated the severity of colons damage and inflammatory infiltration (Scale bar, 100 µm) (**c**). The control group of each experiment shown in Supplementary Fig. 13 d, e, and g. *n* = number of biological replicates. *P* values are calculated by using two-tailed unpaired Student's *t* test (**b**). Error bars represent mean ± s.e.m. Source data are provided as a Source Data file.

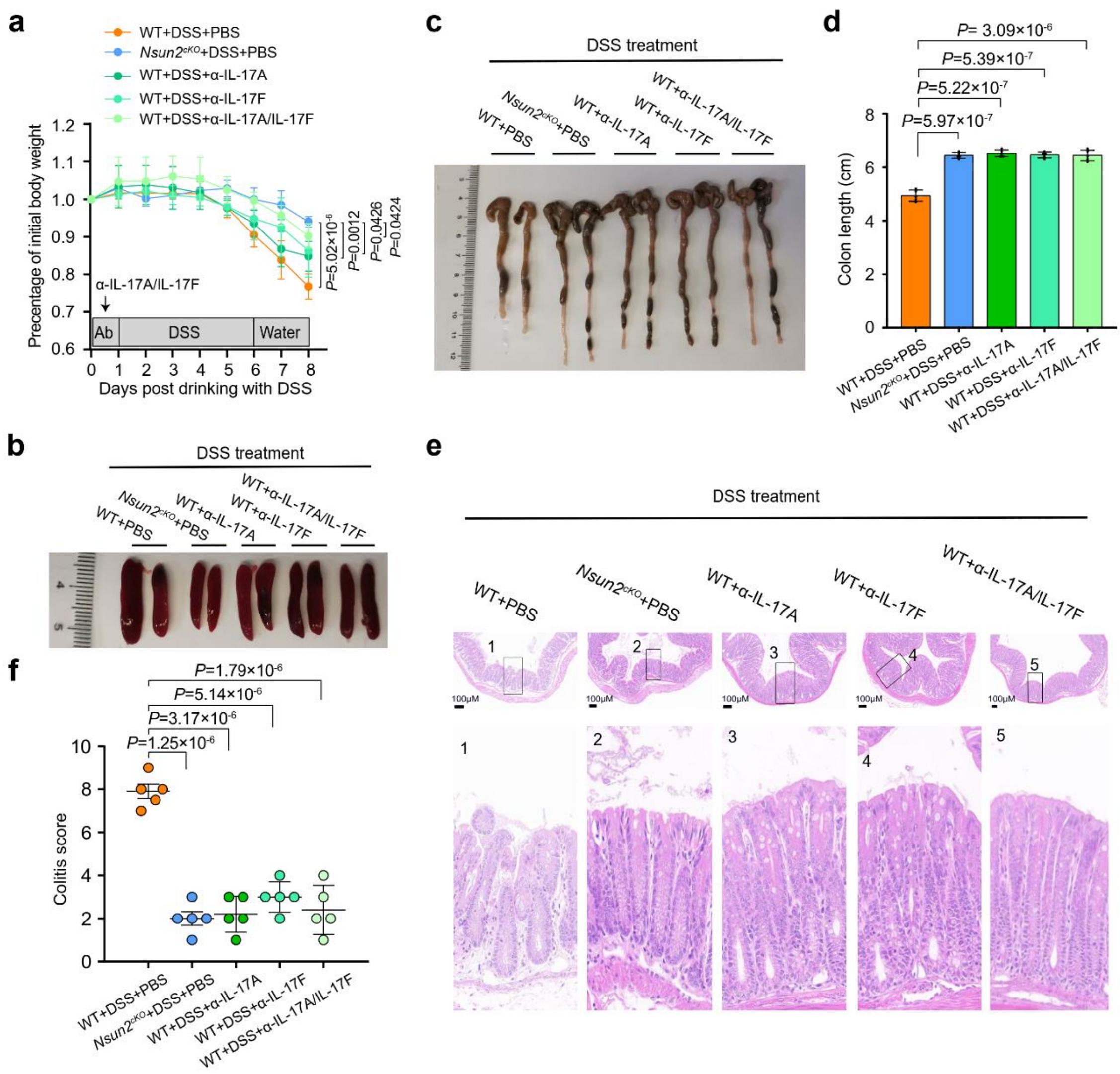

Supplementary Fig. 16 | Neutralization of IL-17A and IL-17F alleviates the colitis development in DSS-induced mice.

a-f, The wild-type (n = 5) and *Nsun2*^{cKO} (n = 5) mice were treated with 3% DSS in drinking water after blockage of IL-17A (250 µg/mouse) or IL-17F (250 µg/mouse) alone, or both IL-17A (250 µg/mouse) and IL-17F (250 µg/mouse) signaling via antibodies. Five mice were used in each group. Body weight changes (**a**), representative macrograph of spleens (**b**) and colons (**c**), the colon length (n = 5 mice per group) (**d**), hematoxylin-eosin staining indicated the severity of colons damage and inflammatory infiltration (Scale bar, 100 µm) (**e**), and colitis score (n = 5 mice per group) (**f**). n = number of biological replicates. P values are calculated by using two-tailed unpaired Student's t-test (**a**, **d and f**). Error bars represent mean ± s.e.m. Source data are provided as a Source Data file.

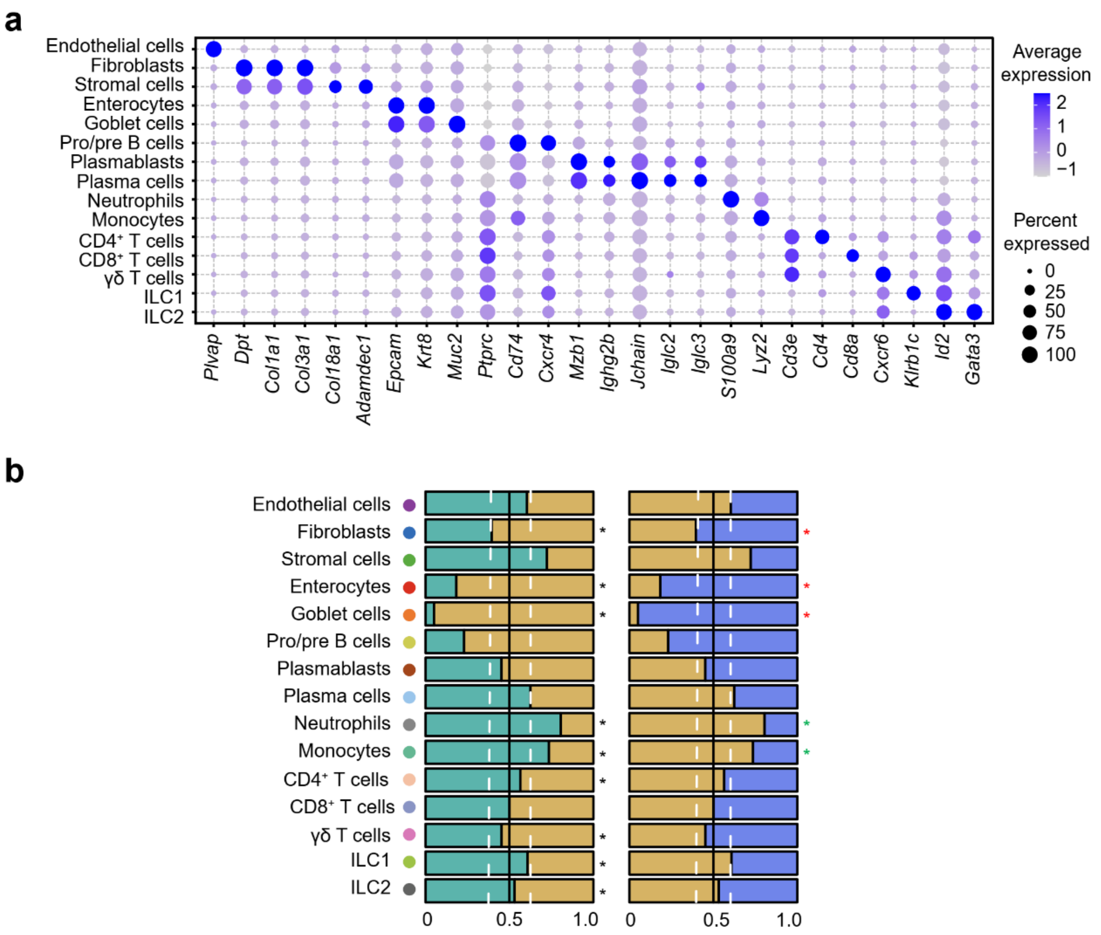

Supplementary Fig. 17 | Characterization of clusters was identified by single cell RNA-seq.

a, Bubble plot showing the average expression and the percentage of expressed cells for markers in each cell type. **b**, Barplots showing the proportion of cell types across WT+H_2O (in Green), WT+DSS (in Gold) and *Nsun2*^{cKO}+DSS treatments. The aberrantly changed cell types during colitis were marked with black asterisk (left). In colitis, the rescued increased cell types upon Nsun2 deletion were marked with red asterisk, the rescued decreased cell types were marked with green asterisk (right).

Supplementary Data

Supplementary Data 1
Protein list for the Nsun2 immunoprecipitation and mass spectrometric analyses.

Supplementary Data 2
A summary of ATAC-seq information in this study.

Supplementary Data 3
A summary of mRNA-seq, caRNA-seq, m^5C MeRIP-seq, and Nsun2 RIP-seq information in this study.

Supplementary Data 4
A summary of single cell RNA-seq information in this study.

Supplementary Data 5
A summary of antibodies and reagents information used in this study.

Supplementary Data 6
A summary of plasmids information in this study.

Supplementary Data 7
A summary of primers and reporter genes sequence information in this study.
