# Supplementary material for: Nsun2 coupling with RoRγt shapes the fate of Th17 cells and promotes colitis
Source: Nat Commun. 2023 Feb 16;14:863. doi: 10.1038/s41467-023-36595-w (PMC9932167; doi:10.1038/s41467-023-36595-w)
Supplement: Supplementary file 3 — Description of Additional Supplementary Files [file 41467_2023_36595_MOESM3_ESM.pdf]

Description of Additional Supplementary Files

File name: Supplementary Data 1
Description: Proteins list for the Nsun2 immunoprecipitation and mass spectrometric analyses.

File name: Supplementary Data 2
Description: A summary of ATAC-seq information in this study.

File name: Supplementary Data 3
Description: A summary of mRNA-seq, caRNA-seq, m^5C MeRIP-seq, and Nsun2 RIP-seq information in this study.

File name: Supplementary Data 4
Description: A summary of single cell RNA-seq information in this study.

File name: Supplementary Data 5
Description: A summary of antibodies and reagents information used in this study.

File name: Supplementary Data 6
Description: A summary of plasmids information in this study.

File name: Supplementary Data 7
Description: A summary of primers and reporter genes sequence information in this study.
